# Tobacco control knowledge and beliefs among healthcare workers in respiratory departments in Fujian Province, China: A cross-sectional study

**DOI:** 10.18332/tid/183606

**Published:** 2024-03-13

**Authors:** Maolin Liu, Pengxiang Huang, Xinying Xu, Zishan Chen, Jinman Zhuang, Yuhang Liu, Shuyan Yang, Xiaoyang Chen, Fei He

**Affiliations:** 1 Department of Epidemiology and Health Statistics, School of Public Health, Fujian Medical University, Fuzhou, China; 2 Department of Pulmonary and Critical Care Medicine, Respirology Medicine Centre of Fujian Province, The Second Affiliated Hospital of Fujian Medical University, Quanzhou, China; 3 Department of Pulmonary and Critical Care Medicine, Jinjiang Municipal Hospital, Quanzhou, China

**Keywords:** smoking cessation, tobacco control, healthcare workers

## Abstract

**INTRODUCTION:**

Smoking prevalence is high in China, and healthcare workers are important for tobacco control. This study aimed to determine the smoking status, cognition of tobacco hazards, and smoking cessation-related knowledge among respiratory healthcare workers, and to explore their ability to provide smoking cessation assistance.

**METHODS:**

A cross-sectional study was conducted in 2021 among 1028 respiratory healthcare workers from 89 hospitals in Fujian Province, China. A self-designed electronic questionnaire was used to collect data on smoking status, knowledge of smoking hazards, and smoking cessation knowledge. Descriptive statistics were calculated for all questions. Logistic regression analysis was used to explore the relationship between awareness of the tobacco control goals of Healthy China 2030 and demographic characteristics.

**RESULTS:**

Among the healthcare workers surveyed, 3.4% were smokers, all of whom were male. Most respondents (99.4%) were aware of smoking as a cause of lung cancer, but awareness of smoking as a cause of non-respiratory cancer was lower. The awareness rate of smoking cessation support was high (>90%), but only 40.0% of participants were aware of the Healthy China 2030 tobacco control targets. Male (HR=2.16; 95% CI: 1.69–2.80) and participation in the cessation clinic (HR=1.47; 95% CI: 1.10–1.96) were associated with higher awareness of the targets.

**CONCLUSIONS:**

Respiratory healthcare workers in Fujian Province demonstrated a high level of awareness regarding behavioral and pharmacotherapy support for smoking cessation. In order to enable healthcare workers to play a more active role in tobacco control, there is a need to increase public awareness of smoking cessation services in Fujian Province.

## INTRODUCTION

The tobacco epidemic is one of the most serious public health threats worldwide. Tobacco use is a major cause of death, illness, and poverty^1^. According to the 2019 Global Burden of Disease Study, the prevalence of smoking among men and women aged ≥15 years was 32.7% and 6.6%, respectively, while in China, the corresponding prevalence rate was 49.7% and 3.5%, respectively. There are an estimated 341 million smokers in China, accounting for 30% of the global total^2^. In Fujian Province, the current smoking prevalence among the population aged ≥15 years is 25.3% (48.9% in males and 0.9% in females)^3^.

Tobacco use is a major risk factor for cardiovascular and respiratory diseases, for over 20 different types or subtypes of cancer, and many other debilitating health conditions^4^. According to the Global Burden of Disease Study, smoking was the leading underlying cause of death in China in 2017, accounting for 2.2 million deaths among smokers; in addition, there were another 0.4 million deaths due to passive smoking. Moreover, smoking was the leading cause of disability-adjusted life-years^5^. Smoking cessation can have immediate and long-term health benefits in people of all ages, due to a reduction in tobacco-related cardiovascular, respiratory, and other systemic diseases. Moreover, the earlier a person quits smoking, the longer the life expectancy he or she can achieve^6^.

The Healthy China 2030 strategy formulated by the Chinese government specifies clear actions and targets for tobacco control: by 2030, the prevalence of smoking among people aged >15 years will be lower than 20%, and the proportion of the population protected by comprehensive smoke-free laws will be ≥80%. Tobacco control is a key component of the Healthy China 2030 plan. In order to achieve this goal, China needs to take strong actions to strengthen the implementation of the WHO Framework Convention on Tobacco Control (FCTC) MPOWER measures: Monitor tobacco use and prevention policies; Protect people from tobacco smoke; Offer help to quit tobacco use; Warn people about the dangers of tobacco; Enforce bans on tobacco advertising, promotion, and sponsorship; and Raise taxes on tobacco. Article 14 of the FCTC requires parties to promote tobacco cessation and implement effective measures to help tobacco users to quit^7^. However, this is poorly implemented, including in China. By 2021, only 26 countries were offering smoking cessation services at the highest level^8,9^.

China has taken significant steps to control tobacco use, in line with the specific goals and requirements set by the government and society. Healthy China 2030 mandates medical staff to abstain from smoking during working hours and encourages counseling and support for patients to quit smoking. Healthcare workers are key players in tobacco control activities, acting as educators and counselors. Respiratory medical staff, in particular, are at the forefront of contacting, persuading, and educating smokers. Furthermore, hospitalization is an opportune time to provide smoking cessation intervention measures for smokers^10^. If respiratory medical staff have a good understanding of tobacco control, they can more effectively persuade smokers to quit smoking and provide appropriate treatment in clinical work and outpatient smoking cessation services, which is highly beneficial to the implementation of Article 14 of the FCTC.

This study examined the smoking status, knowledge of tobacco, and smoking cessation among healthcare workers in the respiratory departments of hospitals in Fujian Province. We also investigated whether they set a good example for the general public, assessed their ability to provide smoking cessation assistance, and explored how healthcare workers in China could better provide smoking cessation assistance.

## METHODS

### Study design

This cross-sectional study aimed to determine the cognition of tobacco and smoking cessation-related knowledge among respiratory healthcare workers in Fujian Province. The inclusion criteria were: 1) in-service staff, and 2) ability to understand and voluntarily participate in the questionnaire survey. The exclusion criteria were: 1) healthcare workers on sick leave, and 2) inability to complete the questionnaire independently. A total of 1028 healthcare workers (doctors and nurses) from respiratory departments of all 89 hospitals at provincial, municipal, and county levels in Fujian Province, China, were enrolled in the study. The response rate was 100%.

### Data collection

The directors of respiratory departments of 89 hospitals in Fujian Province were contacted in advance of the survey to inform them of the survey’s purpose and content and to obtain their understanding and cooperation. From March to April 2021, these directors distributed the digital questionnaire to medical staff who met the inclusion criteria through Questionnaire Star, an online platform (www.wjx.cn). A total of 1028 medical staff participated in the online questionnaire survey, and the questionnaires were valid.

The questionnaire was designed by our team and covered the following topics:

1. Medical staff’s demographic information and tobacco use status.
2. Medical staff’s knowledge of various cancers caused by smoking.
3. Medical staff’s mastery of behavioral support for smoking cessation (using the USPHS Clinical Practice Guideline’s smoking cessation intervention referred to as the 5As).
4. Medical staff’s mastery of pharmacotherapy support for smoking cessation (nicotine replacement therapy, bupropion, and varenicline).
5. Medical staff’s cognition of Healthy China 2030 tobacco control targets (by 2030, the prevalence of smoking among people aged >15 years will be lower than 20%, and the proportion of the population protected by comprehensive smoke-free laws will be ≥80%).

### Statistical analysis

SPSS 26.0 software was used for statistical analysis. Categorical variables were expressed as frequencies and percentages, and between-group differences were assessed using the chi-squared (χ^2^) test. Univariate and multivariable logistic regression analysis were used to explore the relationship between the mastery of Healthy China’s 2030 tobacco control goals and demographic characteristics. Gender, age, education level, medical staff type, professional titles, and whether to participate in a cessation clinic, were included in the multivariable logistic regression analysis model. The forward LR method was used to construct the multivariable logistic regression analysis model, which leads to the adjusted odds ratio (AOR) and confidence limit. The significance was determined as p<0.05 (two-tailed), and the confidence limit was 95%.

## RESULTS

### Characteristics of the study population

A total of 1028 questionnaires were collected in this survey, and the response rate was 100%. The respondents included 276 males (26.8%) and 752 females (73.2%). There were 448 physicians (43.6%) and 580 nurses (56.4%), among which 590 had junior professional titles (57.4%), 268 had intermediate professional titles (26.1%), and 170 had senior professional titles (16.5%).

### Smoking status

Among the 1028 healthcare workers surveyed, 35 were smokers (3.4%). Nineteen were ex-smokers (54.3%), and 16 were current smokers (1.6%). Significant differences in smoking status were observed among healthcare workers according to gender, age, education level, professional title, and working position (p<0.001). The smoking rate of males was 12.7%, while no smokers were found among the females. The higher the age and professional title, the greater the smoking prevalence. Among the healthcare workers with a Bachelor’s degree or above, the higher the education level, the lower the smoking rate (Table 1).

**Table 1:** Smoking status of healthcare workers in respiratory departments in Fujian Province, 2021 (N=1028)

*Characteristics*	*Non-smokers n (%)*	*Ex-smokers n (%)*	*Current smokers n (%)*	*χ^2^*	*p*
**Gender**				98.724	<0.001
Male	241 (87.3)	19 (6.9)	16 (5.8)		
Female	752 (100)	0 (0.0)	0 (0.0)		
**Age** (years)				26.607	<0.001*
20–34	662 (98.5)	3 (0.4)	7 (1.0)		
35–49	286 (93.5)	12 (3.9)	8 (2.6)		
50–59	45 (90.0)	4 (8.0)	1 (2.0)		
**Education level**				22.774	<0.001*
Junior college or lower	403 (99.5)	0 (0.0)	2 (0.5)		
Undergraduate	444 (94.3)	16 (3.4)	11 (2.3)		
Postgraduate or higher	146 (96.1)	3 (2.0)	3 (2.0)		
**Professional title**				28.095	<0.001*
Junior	583 (98.8)	2 (0.3)	5 (0.8)		
Intermediate	256 (95.5)	7 (2.6)	5 (1.9)		
Senior	154 (90.6)	10 (5.9)	6 (3.5)		
**Medical staff type**				42.417	<0.001
Physician	414 (92.4)	19 (4.2)	15 (3.3)		
Nurse	579 (99.8)	0 (0.0)	1 (0.2)		
**Cessation clinic**				2.073	0.355
Yes	363 (95.8)	10 (2.6)	6 (1.6)		
No	630 (97.1)	9 (1.4)	10 (1.5)		

* Fisher’s exact test.

### Cognition of tobacco harm

**Figure 1:**
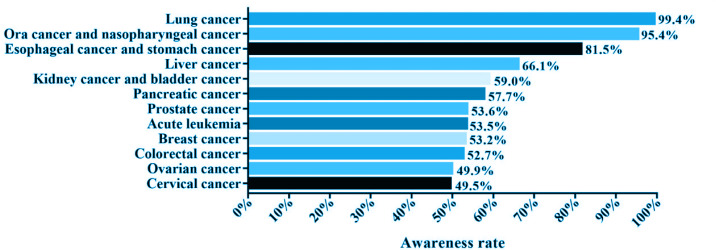
Awareness rate of various types of cancer caused by smoking among medical staff in respiratory departments in Fujian Province, 2021 (N=1028)

Among the 1028 respondents, the highest awareness rate of various cancers caused by smoking was for lung cancer (99.4%), followed by oral and nasopharyngeal malignancies (95.4%), while the awareness rate of non-respiratory tumors caused by smoking was relatively low. The type of cancer that was least known to be caused by smoking was cervical cancer (49.5%) (Figure 1).

### Mastery of behavioral and pharmacotherapy support for smoking cessation

The awareness rate of all smoking cessation behavioral support was more than 95%, and the awareness rate of all pharmacotherapy support was more than 90% (Table 2).

**Table 2:** The cognition of behavioral and pharmacotherapy support for smoking cessation among healthcare workers in respiratory departments in Fujian Province, 2021 (N=1028)

*Support for smoking cessation*	*n*	*%*
**Behavioral**		
Ask about tobacco use	1011	98.3
Advise tobacco users to quit	1013	98.5
Assess willingness to make a quit attempt	1007	98.0
Assist tobacco users in making a quit attempt	998	97.1
Arrange for follow-up	986	95.9
**Pharmacotherapy**		
Nicotine replacement therapy	993	96.6
Bupropion	960	93.4
Varenicline	938	91.2

### Cognition of the Healthy China 2030 tobacco control targets

Only 40.0% of the respondents were aware of the Healthy China 2030 tobacco control targets. The awareness of the Healthy China 2030 tobacco control targets varied according to gender, age, education level, medical staff type, professional title, and whether to participate in a cessation clinic (Table 3). Male physicians who were older, had higher education and higher professional titles and participated in a smoking cessation clinic had a relatively higher awareness of the Healthy China 2030 tobacco control targets.

**Table 3:** Univariate logistic regression analysis of factors affecting the cognition of the Healthy China 2030 tobacco control targets, among medical staff in respiratory departments in Fujian Province, 2021 (N=1028)

*Variables*	*Aware of the tobacco control targets for Healthy China 2030 n (%)*	*OR (95% CI)*	*p*
**Gender**			
Female ®	274 (36.4)	1	
Male	137 (49.6)	1.72 (1.30–2.27)	<0.001
**Age** (years)			
20–34 ®	250 (37.2)	1	
35–49	135 (44.1)	1.33 (1.01–1.75)	0.040
≥50	26 (52.0)	1.83 (1.03–3.25)	0.040
**Education level**			
Junior college or lower ®	152 (37.5)	1	
Undergraduate	188 (39.9)	1.11 (0.84–1.45)	0.470
Postgraduate or higher	71 (46.7)	1.46 (1.00–2.13)	0.049
**Medical staff type**			
Nurse ®	209 (36.0)	1	
Physician	202 (45.1)	1.46 (1.13–1.86)	0.003
**Cessation clinic**			
No ®	196 (51.7)	1	
Yes	215 (33.1)	2.16 (1.67–2.80)	<0.001
**Professional titles**			
Junior ®	211 (35.8)	1	
Intermediate	114 (42.5)	1.33 (0.99–1.79)	0.058
Senior	86 (50.6)	1.84 (1.30–2.60)	0.001

® Reference categories.

Gender, age, education level, medical staff type, professional title, and whether to participate in a cessation clinic were included in the multivariable logistic regression analysis model. The results of the multivariable logistic regression analysis showed that men had a higher awareness rate of the Healthy China 2030 tobacco control targets (AOR=2.16; 95% CI: 1.69–2.80) than women. Additionally, the awareness rate of medical staff participating in the smoking cessation clinic (AOR=1.47; 95% CI: 1.10–1.96) was higher than those who did not participate in the smoking cessation clinic (Table 4).

**Table 4:** Multivariable logistic regression analysis of factors affecting the cognition of the Healthy China 2030 tobacco control targets, among medical staff in respiratory departments in Fujian Province, 2021 (N=1028)

*Characteristics*	*β*	*SE*	*Wald χ^2^*	*AOR (95% CI)*	*p*
**Gender**					
Female ®				1	
Male	0.771	0.132	33.929	2.16 (1.69–2.80)	<0.001
**Cessation clinic**					
No ®				1	
Yes	0.383	0.147	6.767	1.47 (1.10–1.96)	0.009

AOR: adjusted odds ratio; adjusted for gender, age, education level, medical staff type, professional titles, and whether to participate in cessation clinic. ® Reference categories.

## DISCUSSION

In this study, the smoking rate among respiratory healthcare workers in Fujian Province was 3.4%, with 12.7% of males and 0% of females being smokers. This rate was significantly lower than that of the general population in Fujian Province. According to the 2018 WHO Global Adult Tobacco Survey, the current smoking rate among Chinese healthcare workers was 14.2% (37.9% in males and 0.6% in females), and 20.5% of healthcare workers who used to smoke had quit smoking successfully^11^. The current smoking rate of respiratory healthcare workers in our study was 1.6%, with 5.8% of males and 0% of females being smokers. This rate was significantly lower than that of Chinese healthcare workers. The smoking cessation rate was 54.3%, which was significantly higher than the smoking cessation rate of Chinese medical workers. Healthcare workers play an important role in tobacco control, and their own smoking behavior directly affects the attitudes and behavior of smokers toward tobacco cessation^12^. The low smoking rate and high cessation rate of healthcare workers in respiratory departments of Fujian Province may be attributed to their professional knowledge and clinical experience. Their experience in smoking control can be used for reference by medical staff in other departments, and they can also serve as a good example for social smokers.

The 2019 Global Burden of Disease Study revealed that tobacco is the leading Level 2 risk factor for deaths for males globally^13^, resulting in 7.69 million deaths and 200 million disability-adjusted life-years worldwide^2^. The ill effects of smoking on health have long been established. In this study, the awareness rate of various cancers caused by smoking among 1028 respiratory healthcare workers was investigated. The awareness rate of smoking as a cause of lung cancer, oral malignancy, and nasopharyngeal malignancy was high; however, there was low awareness of smoking as a cause of non-respiratory malignancies, such as digestive system, reproductive system malignancy, and acute leukemia. Less than half of healthcare workers were aware that smoking causes cervical and ovarian cancer. To better educate and persuade smokers and help them to quit smoking, it is essential to strengthen the cognition of respiratory healthcare workers in Fujian Province regarding the hazards of smoking. Compared with the general population, healthcare workers are exposed to more health-related information, but their cognition of the harm of smoking is still limited, which reflects the limited cognition among the general population. Cognition of the harm of smoking was shown to have an important influence on smoking behavior and the willingness of smokers to quit smoking^14^. Increasing public awareness of the hazards of smoking improves compliance with regulations related to smoking in public places and encourages changes in the smoking behavior of smokers.

In the period 2016–2017, the smoking cessation rate of adult smokers in Fujian Province was 15.1%^15^. Apart from a lack of willpower and self-discipline, family support, social activities, and professional life also play an important role in smoking cessation^16^. The USPHS Clinical Practice Guideline recommends that health workers provide a brief, evidence-based smoking cessation intervention referred to as the 5As: Ask about tobacco use; Advise tobacco users to quit; Assess willingness to make a quit attempt; Assist tobacco users in making a quit attempt; and Arrange for follow-up^17^. The use of 5As smoking cessation intervention and nicotine replacement therapy, bupropion, varenicline, and other drugs to help smokers quit smoking have been proven to be effective^18-20^. The awareness rate of smoking cessation intervention measures and smoking cessation-related drugs among respiratory healthcare workers in Fujian Province was higher than 90%, indicating that they have the ability to provide good help for smokers who want to quit smoking, both in the clinical process and in smoking cessation outpatient services.

A study found that 31.8% of smokers in China were considering quitting smoking^21^, and if these people succeed in quitting, China can easily achieve the Healthy China 2030 tobacco control target. However, smokers typically quit alone and rarely seek help from smoking cessation outpatient services. The establishment and development of smoking cessation outpatient services in China have achieved certain results, but its development is still facing many problems, such as the low utilization rate of smoking cessation drugs and the uneven distribution of smoking cessation clinics. The most important problem is the need to improve service utilization of smoking cessation clinics^22^. Despite the fact that many smokers were willing to quit smoking, the awareness rate of smoking cessation outpatient services in the general population was found to be very low. A study found that a very low proportion of smokers who were willing to quit smoking in Fujian Province sought help. Among those who quit, only 1.5% used drugs to quit smoking, 0.5% of them received outpatient counseling, and none of them used the smoking cessation hotline^15^. To improve the utilization of smoking cessation services, it is essential to raise awareness among the general population about the importance of seeking professional help for quitting smoking.

Due to the large number of patients in China, the short duration of outpatient treatment, and the large number of patients during hospitalization, it is difficult for Chinese healthcare workers to provide long-term counseling and follow-up for smokers in clinical practice. Studies have shown that Chinese healthcare workers are more likely to provide brief smoking cessation interventions in the busy clinical process^23^. Although brief smoking cessation interventions can have a certain effect on smoking cessation^24^, quitting smoking is a difficult process and typically entails multiple attempts. Therefore, the long-term companionship of healthcare workers during the process of smoking cessation is very important^25^. In a study, simple active referrals (inperson or via text messaging) to smoking cessation services were found to increase abstinence rates among smokers compared with general brief cessation advice and increased the use of smoking cessation services^26^. However, more than half of the respiratory medical healthcare workers in Fujian Province were not clear about the Healthy China 2030 tobacco control target, which is not conducive to their role in tobacco control. It is essential to raise the awareness of tobacco control targets among healthcare workers so that they can more actively recommend smokers to seek smoking cessation help through smoking cessation outpatient services during clinical diagnosis and treatment. This would not only increase the use of smoking cessation clinic resources but also provide long-term and effective smoking cessation interventions that are better placed to help smokers quit smoking. Additionally, the Chinese society and government should work together to raise awareness among the general population about the importance of seeking professional help for quitting smoking. This would improve the utilization of outpatient smoking cessation services, help optimize the use of medical resources, and enable more smokers to benefit from the assistance of healthcare workers.

### Limitations

Our study conducted a questionnaire survey among all respiratory healthcare workers working at hospitals at all levels in Fujian Province, without sampling bias, to objectively and comprehensively analyze the ability of respiratory healthcare workers of Fujian Province to provide smoking cessation assistance. However, there are some limitations to consider when interpreting the results. Firstly, the study design was a cross-sectional study, meaning that it could not establish a cause-and-effect relationship between the variables studied. There may be residual confounding in the study, which refers to the presence of unmeasured or unknown factors that may have influenced the results. Secondly, our study only included healthcare workers in respiratory departments, examined their cognitive status of smoking cessation-related knowledge, and evaluated their ability to provide help for smoking cessation, but did not study the actual implementation of smoking cessation intervention provided by them in clinical practice and smoking cessation outpatient services. Additionally, the study’s findings may have limited generalizability to other countries, as the study was conducted only in Fujian Province. Finally, our study did not examine the awareness of health workers regarding non-traditional modes of tobacco use, such as e-cigarettes. In recent years, electronic cigarettes have been proposed as a method for smoking cessation by acting as a substitute for cigarettes. Although e-cigarettes were shown to be less harmful than traditional cigarettes^27^, they are also harmful to the human body^28^. E-cigarettes are becoming increasingly popular, and there is a temporary lack of corresponding control measures and industry management policies. Therefore, it is important to study the attitudes of healthcare workers towards e-cigarettes in order to formulate better tobacco control measures.

## CONCLUSIONS

The smoking rate among respiratory healthcare workers in Fujian Province was low, and the smoking cessation rate was high, indicating that these healthcare workers are setting a good example for their peers and society. They also had a high awareness rate of behavioral and pharmacotherapy support for smoking cessation. However, there is a need to improve their knowledge related to the harms of smoking and the causes of failure to quit smoking, so that they can provide better smoking cessation services. In general, the respiratory healthcare workers in Fujian Province have the ability to provide good smoking cessation help for smokers. To ensure that more smokers can benefit from quitting smoking, the government should take more steps to improve the accessibility and quality of smoking cessation services.

## Data Availability

The data supporting this research are available from the authors on reasonable request.
